# Supplementary figures and images for: Identifying key genes in COPD risk via multiple population data integration and gene prioritization
Source: PLoS One. 2024 Nov 7;19(11):e0305803. doi: 10.1371/journal.pone.0305803 (PMC11542775; doi:10.1371/journal.pone.0305803)

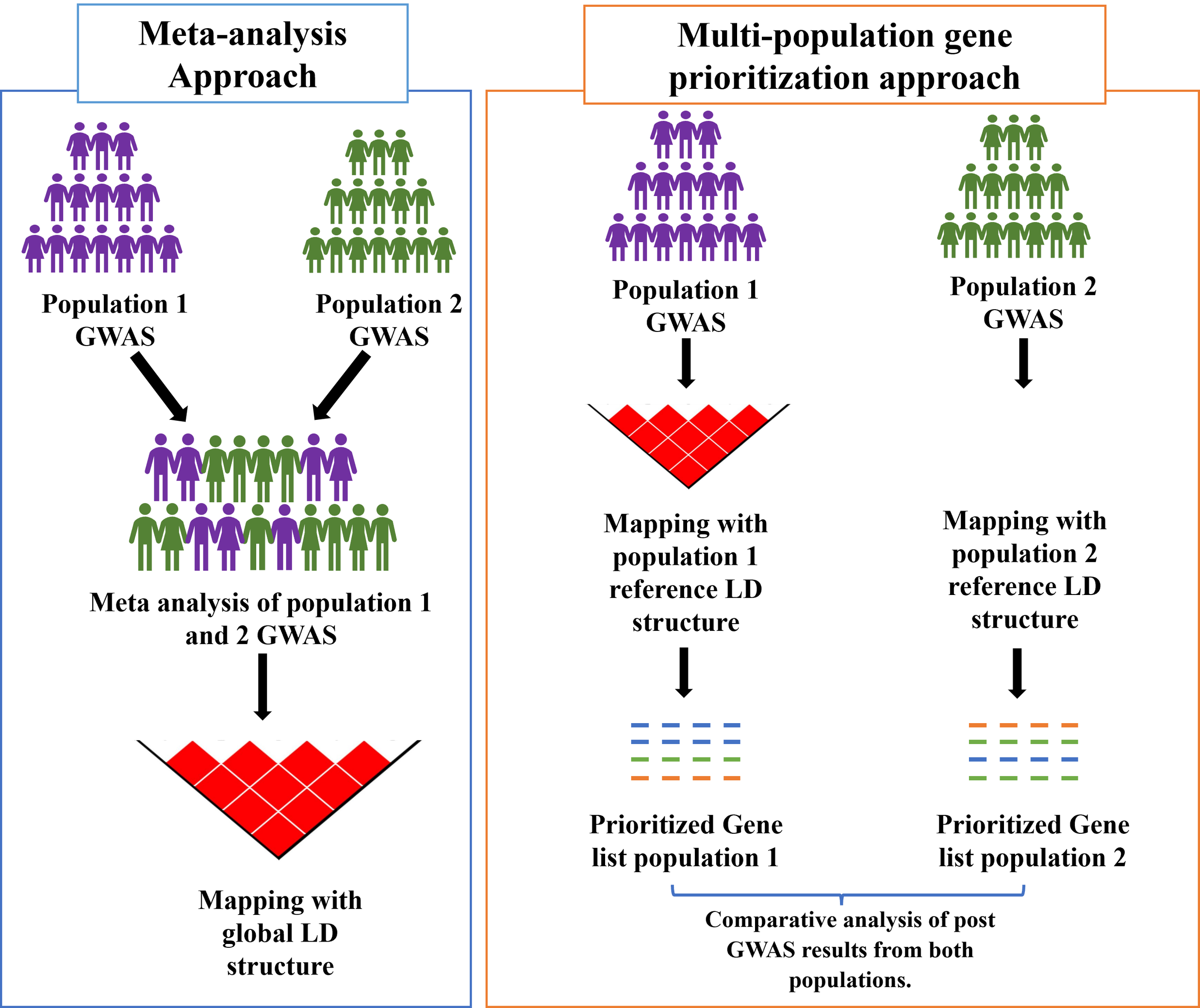

Supplement: S1 Fig — The meta-analysis approach (left) works by combining GWAS results from two populations (purple and green groups) into a single study through a merged linkage disequilibrium (LD) structure. The multi-population gene prioritization approach (right) keeps distinct GWAS and unique LD structures for each population for further analysis. (TIF) [file pone.0305803.s001.tif]

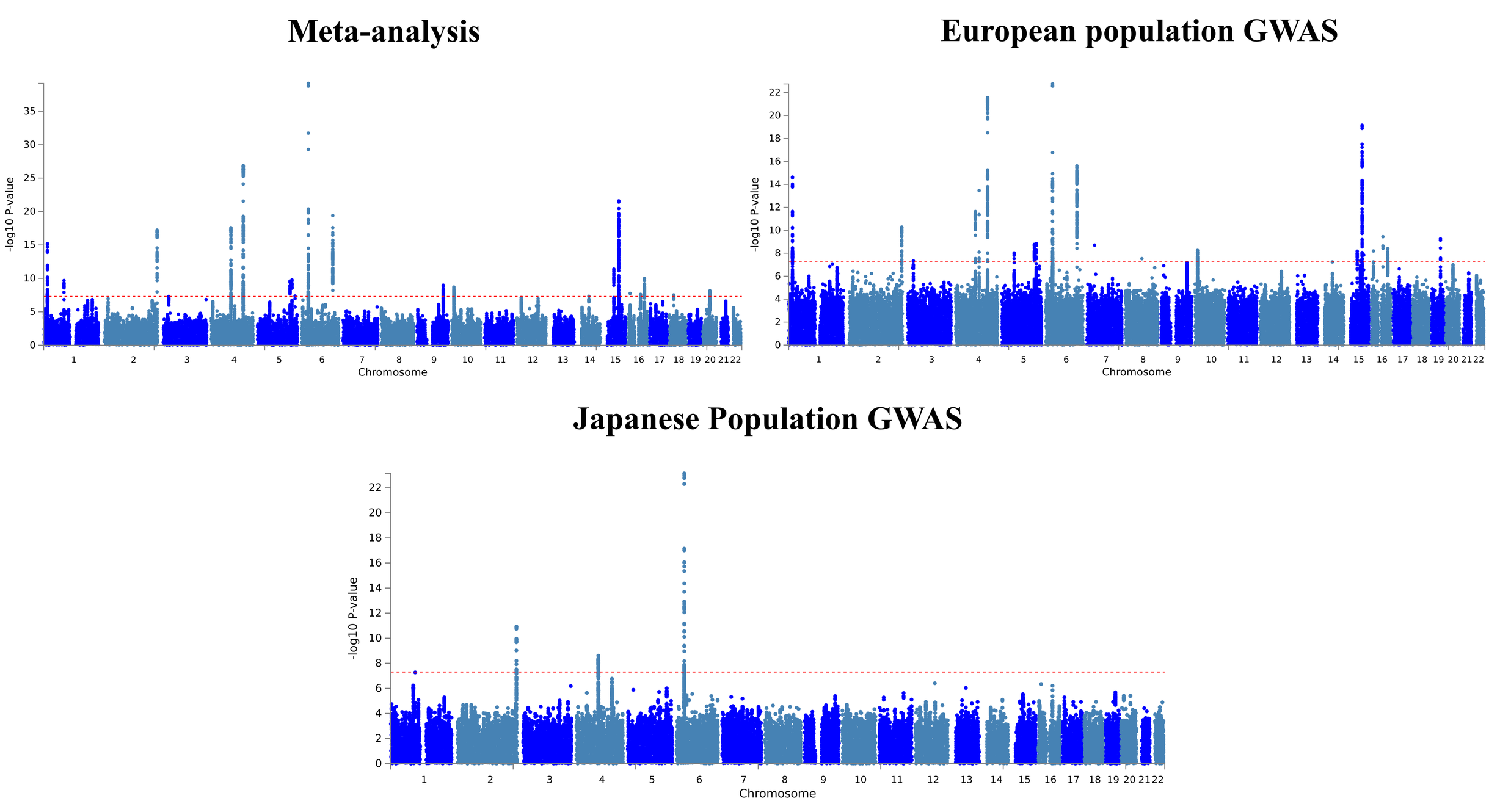

Supplement: S2 Fig — According to the meta-analysis (top-left) stronger signals are particularly seen at chromosomes 3, 4, and 17, While the Japanese GWAS (bottom) shows peaks on chromosomes 3 and 4, the European GWAS (top-right) highlights significant peaks on chromosomes 3, 5, and 17 (similar to meta-analysis). The genome-wide significance threshold is indicated by red dashed lines, which show how association signals vary among different populations. (TIF) [file pone.0305803.s002.tif]

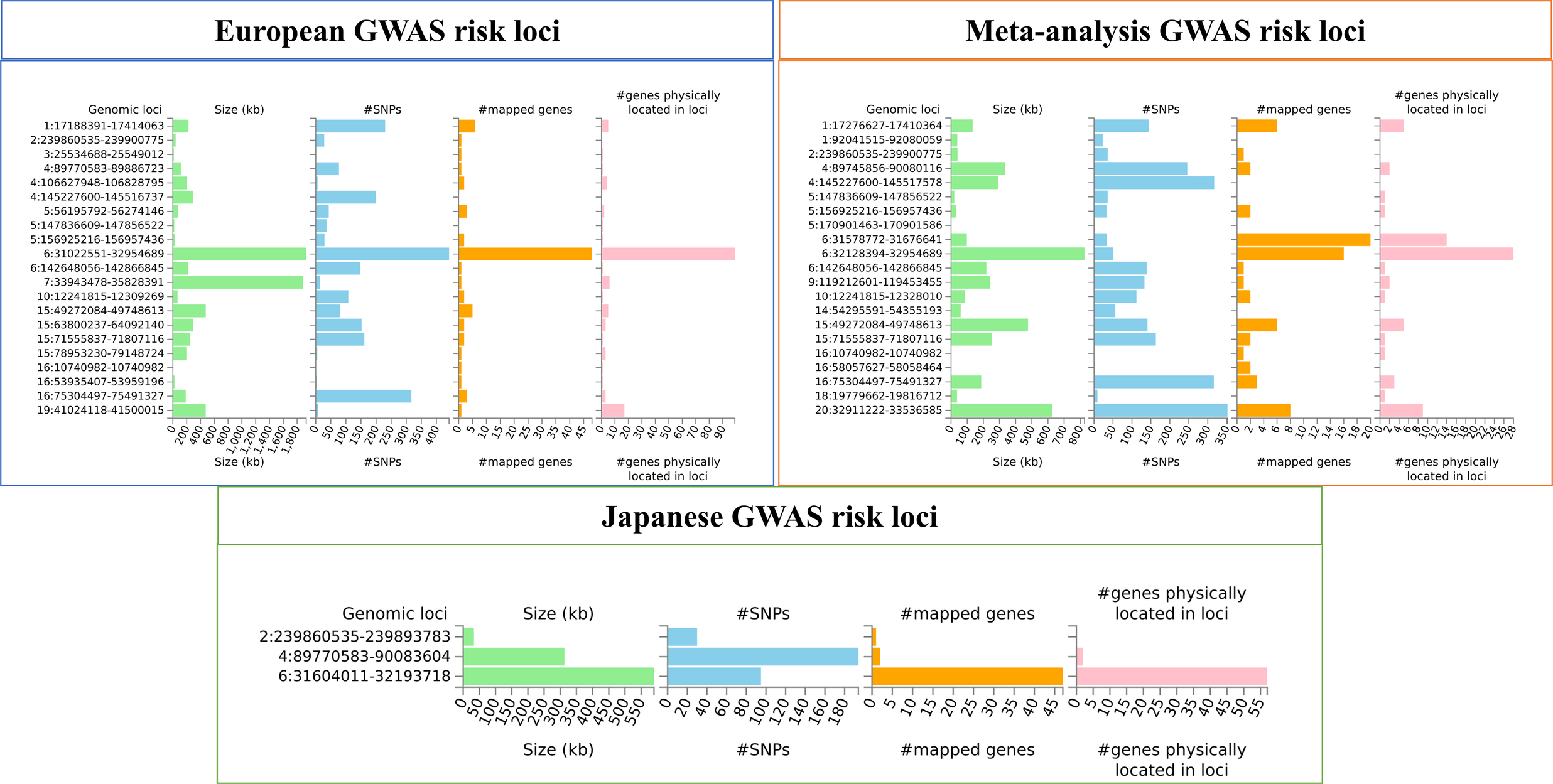

Supplement: S3 Fig — The y-axis shows the size of loci in kb, the number of SNPs in the loci, genes mapped within the risk loci, and a number of genes physically located in loci. (TIF) [file pone.0305803.s003.tif]
