# Supplementary material for: Identifying key genes in COPD risk via multiple population data integration and gene prioritization
Source: PLoS One. 2024 Nov 7;19(11):e0305803. doi: 10.1371/journal.pone.0305803 (PMC11542775; doi:10.1371/journal.pone.0305803)
Supplement: Attachment — Submitted filename: Response to Reviewers.docx [file pone.0305803.s009.docx]

**Specific Comments by Reviewers**

**Reviewer 1**

Thank you for taking the time to review our manuscript; we have incorporated the suggested changes and updated it. Kindly find detailed responses to individual comments as follows:

**Comment 1:** In this study, three types of mapping approaches have been used to identify and prioritize the genes, with one of them as chromatin interaction mapping. In line 175, the authors stated that no genes were mapped as a result of chromatin interaction mapping in the Japanese population, while two genes have been identified in the European population. The authors should discuss the potential reasons for not identifying the genes in the Japanese population. In Fig. 1, the results of chromatin interaction mapping in the Japanese population should also be illustrated.

- **Response:** There can be several reasons for the lack of results in the Japanese population for chromatin interactions. Evolutionary changes and environmental factors can cause population-specific changes in the chromatin landscape. These factors can change epigenetic profiles, affecting gene expression and altering chromatin interaction signals. Furthermore, The Japanese population may have smaller effect sizes due to differences in different LD structures, which may not meet the strict statistical thresholds (e.g. FDR < 1e−6, as suggested by Schmitt et al. *Cell Rep*, 2016) required to identify significant interactions. More interactions might become visible if these thresholds are lowered.
   We have added descriptions in **Lines 212-223** of the revised manuscript to clarify these points. To make the comparison with the European results more transparent, we have also updated Figure 1 to incorporate the chromatin interaction mapping results for the Japanese population. With these revisions, we think the reviewer's concerns are addressed, and a more thorough explanation is provided.

**Comment 2:** In Table 1, the Japanese population (N=14061) exhibits a significantly lower number of genomic risk loci, lead SNPs, independent SNPs, and candidate SNPs compared to the European population (N=79055). This discrepancy raises the question of whether the disparity is mainly due to differences in sample size or if other factors also contribute. The authors should discuss it comprehensively.

- **Response:**   Increased statistical power from a larger sample size facilitates the detection of relationships, particularly those with smaller effect sizes, which can be partly responsible for the discrepancy in the number of genomic risk loci, lead SNPs, and independent SNPs found. However, there can also be other factors that influence this variation. For example, certain variations may be easier to find in one population than another due to reasons such as variations in genetic diversity, linkage disequilibrium LD patterns, and allele frequencies among populations, which might affect the identification of loci and SNPs, in addition to sample size. Therefore, our approach also focused on the LD structures of each population.
  To clarify this point, we have added details and discussions to address your comment from **lines 257-271**.

**Comment 3:** In lines 234-236, the authors stated that they performed pathway analysis regarding 28 genes common to both populations. In Fig. 3, the results showed that the number of genes enriched for each pathway was around 2-3. The interactions among these genes were not illustrated thoroughly. Since the 28 genes are all protein-coding genes, protein-protein interaction network analysis should also be performed to better understand the functional relationships of genes in shared biological processes.

- **Response:** Thank you for the excellent suggestion to include protein-protein interaction (PPI) network studies for the 28 frequent protein-coding genes. We conducted a PPI network analysis and included the results and description in the revised manuscript from **lines 337-377,** along with Fig 4 illustrating the PPI network generated using the STRING database. This added functional significance to the existing results as the relationship between the prioritized genes could be more apparent, which could help understand their mutual interactions.

Comment 4: In line 244, the authors stated that the enrichment analysis was solely based on KEGG pathway. In general, the results of GO terms should also be provided. In addition, Supplementary Fig4 (line 245) was mistakenly cited as “the plot illustrating the top KEGG pathways associated with these genes”. The authors should correct it.

- **Response:** To clarify, we have added the findings of the Gene Ontology (GO) term enrichment analysis in the updated manuscript. The supplementary file (S4 file) and **lines 318-319** contain a detailed explanation of these data, enabling a more thorough comprehension of the molecular activities, biological processes, and cellular components related to our results. It also gave significant insights about the genes in terms of regulating the immune system, such as positive regulation of the immune system, positive regulation of T cell activation, and regulation of cell-cell adhesion (S4 file, Table S3) can contribute to inflammation of lung diseases like asthma, chronic obstructive pulmonary disease (COPD), and other respiratory conditions. We think that this addition improves the analysis as a whole and successfully considers your feedback. We have also formatted the mistake in **line 315**.

**Comment 5:** For Figures S1 and S2, the authors should provide detailed descriptions and explanations in the figure legends.

- **Response:** In supplementary information, we have added detailed descriptions for Figures S1 Fig and S2 Fig in **lines 727-737**.

**Comment 6 and other technical comments**
**Line 227: “The analysis showed that populations show” should be “The analysis showed that the populations exhibited”**
**Line 238: “Hydrolysis” should be “hydrolysis”
Line 245: “Genes were found” should be “The genes were found”**
**Line 246: “which indicates” should be “, indicating”; “affect” should be “effects”; “thus” should be “and”.
Line 252: “SNP” should be “SNPs”**

**Line 227: “The analysis showed that populations show” should be “The analysis showed**

**Line 238: “Hydrolysis” should be “hydrolysis”**

**Line 245:** **“Genes were found” should be “The genes were found”**

**Line 246: “which indicates” should be “, indicating”;
“affect” should be “effects”;
“thus” should be “and”.**

**Line 257-259: Should be changed into “There are several ways to perform post-GWAS. In this study, we performed cross-population post-GWAS to identify and prioritize significant genes and SNPs in each population, as well as to identify new independent significant SNPs and important phenotype-associated pathways and genes."**

**Line 263: “many” should be “both”**

**Line 280: "the regulatory enzyme" should be “the regulatory enzymes”, "which synthesize NO" should be "which synthesizes NO"**

**Line 287: “****There's” should be “There has”**

**Line 334: “SNPs” should be “SNP”**

**Line 335-337: “We discovered…the European population GWAS.” should be “We discovered that a novel independent significant SNP, rs137893789, was mapped with TNXB with a CADD score of 15.21 in the European population GWAS using an LD-based specific population approach.”**

**Line 358-359: “Our study…mechanisms.” should be: “In this study, we utilized GWAS data, which is a commonly used method to identify risk-associated variants that play a crucial role in understanding complex genetic mechanisms.**

**Line 368: “same” should be “the same”
Line 370: “underlaying” should be “underlying”
Line 385: “Lead” should be “lead”**

- **Response:** All the above points are revised accordingly.

**Reviewer 2:**

Thank you for reviewing our manuscript and providing valuable feedback. We have provided a detailed explanation of each comment and incorporated the necessary changes.

**Major comments**

**- The resolution of figures is low and it is hard to read the plot text. This is particularly true for Figure 1**

- **Response:** We have updated the figures with high resolution.

**- Please include a description of the model used for GWAS in each cohort, and whether they are directly comparable**

- **Response:** The models were comparable as both studies utilized linear mixed models implemented in BOLT-LMM. The European and Japanese population GWAS outcomes are directly comparable since the same statistical methodology was applied to both cohorts. Because any variation can be more positively attributed to variations in genetic architecture, LD structure, or allele frequencies rather than to differences in analysis methodology, this consistency enables a more trustworthy interpretation of the differences in detected genetic associations between the two populations.

In the revised manuscript, we have clarified the description of the models used for GWAS in each cohort, specifically in **lines 97-101**. We believe these additions make the model comparison more transparent and address your point.

**- L120: is there a reason to choose a specific threshold of “CADD > 12.37”?**

- **Response:** The reason why we used CADD > 12.37 is that it is the minimum threshold for pathogenic SNPs and has been used as a threshold for highly deleterious SNPs. The variant is more likely to have a functional impact than variants with lower scores because it ranks in the top 1% of all scored variants. As in this study, our focus was on functional variants, and this threshold worked well. To clarify this point, we have added the following description in **lines 130-136.**

**- L129: is only the lung data used?**

- **Response:** Yes, only the lung data was used.

**- Several prioritized genes encode the MHC Class II molecules and are among the most polymorphic regions of the human genome. There have been GWAS studies that simply removed the MHC region. I believe this won’t affect the author’s results too much, but I wonder whether the author can comment on this, particularly how transethnic comparison may be affected by the MHC.**

- **Response:** We agree that the MHC region can be excluded in most cases because of its highly polymorphic nature, but we tried to check how it affected the results. As a result, we noticed that excluding the MHC region can miss some crucial immune signals associated with lung function. For example, AGER is one of the genes related to lung function, and it is removed after excluding the MHC region. As a result, the effects of removing the MHC have to be evaluated in light of the particular study questions and characteristics under investigation. To clarify this point, we have added the following description in **lines 149-155**.

**-L227-228: “The analysis showed that populations show genetic diversity, as different SNPs were 228 identified for the same gene in different populations” I think this is a very interesting observation. Is there previous work that makes similar findings?**

- **Response:** Previous studies, such as those analyzing genome-wide association studies (GWAS) across different ancestries, have found that specific genetic variants or SNPs that are linked to a trait in one population may not be linked in another or completely different SNPs within the same gene may emerge as significant.
   One example is for complex diseases like type 2 diabetes, where SNPs in genes like *TCF7L2* are risk factors in various populations. In African Americans, Haddad et al. (*PLoS One* 2017) discovered a novel signal in SNP rs114770437 unrelated to the index SNP rs7903146. The variable rs114770437 has not been detected in prior GWAS of European and Asian ancestry people, which can be explained by the fact that it is monomorphic in the 1000 Genomes Project, which included populations from Europe, East Asia, and South Asia ( *J Hum Genet* 2018). These differences show population-specific evolutionary pressures and underlying genetic diversity.

**-Does this attribute to the genetic background different, or potentially suggest different pathogenesis between populations?**

- **Response:** Different genetic backgrounds among populations may be reflected in the variations in SNP relationships for the same gene. The way that alleles of various SNPs associate, known as linkage disequilibrium (LD) patterns, which was the focus of our study, might vary throughout populations, which can affect which variants are found in association studies. For instance, a particular variant in LD has a causal SNP in one population but not another. These genetic variations might also point to different biological processes or disease pathways in other ethnic groups. Although a gene may contribute to the same disease in various populations, its involvement in altering the mechanism of the disease may differ.
  The impact of genetic polymorphisms on disease risk may be influenced by gene-environment interactions that vary throughout groups, such as dietary, lifestyle, or environmental exposure changes. For example, in communities with varying dietary practices, variations in genes related to metabolism may exhibit distinct relationships with metabolic diseases.
  In summary, these variations highlight how crucial it is to carry out GWAS in various populations since the results can direct the creation of specialized treatment strategies and population-specific risk prediction. Knowing how distinct gene variants affect illness risk in multiple populations is essential for precision medicine. It can aid in discovering new treatment targets, which is also one of the significance of our study.

**- L277: This paragraph (and the later few as well) should belong to the discussion**

- **Response:** As the results and discussion are combined under one heading, we added discussion along with each result.

**- Code availability**

- **Response:**

We have added a detailed description of data analysis steps in the supplementary **S5 file** and a description of the code and data in the code availability section. Furthermore, we added the reference in the methods section from **lines 176-180** as follows:

A meta-analysis was also performed using METAL (cited in the manuscript). The code link can also be found on GitHub as follows: <https://github.com/statgen/METAL.git>

**Minor comments:**

**- While interchangeable, I believe “SNP” is more often used for GWAS, while SNV is usually used for somatic variants in cancer studies.**

- **Response:** We have formatted SNV to SNP.

**- Please make sure to use a consistent format for “trans-ethnic” or “transethnic”**

- **Response:** We have noted and corrected it.
